# RESISTANCE TRAINING EXERCISE FOR THE REHABILITATION OF OLDER ADULTS: A FUNCTION PROMOTING THERAPY WITH ROBUST TREATMENT RESPONSE HETEROGENEITY

**DOI:** 10.1093/geroni/igae098.0939

**Published:** 2024-12-31

**Authors:** Roger Fielding

**Affiliations:** Jean Mayer USDA Human Nutrition Research Center on Aging at Tufts University, Boston, Massachusetts, United States

## Abstract

Advancing age is accompanied by well described reductions in muscle strength and power that are associated with deficits in physical functioning and mobility limitations. Both high and slow velocity progressive resistance exercise training have been shown to improve muscle strength and physical functioning in older adults. In combination with aerobic exercise, resistance exercise can also reduce the risk of major mobility disability. However, despite the overall positive effects exerted by exercise on multiple domains of health and physical functioning, a robust response heterogeneity to exercise training in older adults has been consistently observed. Factors influencing exercise response heterogeneity (ERH) include exercise adherence, sex, age, comorbid conditions, baseline levels of strength and function, and unknown biological factors. Future studies need to interrogate the underlying biological mechanisms associated with ERH. As plausible mechanisms emerge, more personalized approaches may be tailored to optimize the beneficial effect of exercise for all older adults.

